# Integrative analysis of DNA methylation, mRNAs, and small RNAs during maize embryo dedifferentiation

**DOI:** 10.1186/s12870-017-1055-x

**Published:** 2017-06-15

**Authors:** Hongjun Liu, Langlang Ma, Xuerong Yang, Lin Zhang, Xing Zeng, Shupeng Xie, Huanwei Peng, Shibin Gao, Haijian Lin, Guangtang Pan, Yongrui Wu, Yaou Shen

**Affiliations:** 1 Key Laboratory of Biology and Genetic Improvement of Maize in Southwest Region, Maize Research Institute, Sichuan Agricultural University, Chengdu, 611130 China; 2 State Key Laboratory of Crop Biology, College of Life Sciences, Shandong Agricultural University, Tai’an, 271018 China; 3 Department of Agronomy, Northeast Agricultural University, Harbin, 150030 China; 4 Suihua Sub-academy, Heilongjiang Academy of Agricultural Sciences, Suihua, 152052 China; 5 Institute of Animal Nutrition, Sichuan Agricultural University, Ya’an, 625014 China; 6 National Key Laboratory of Plant Molecular Genetics, Institute of Plant Physiology & Ecology, Shanghai Institutes for Biological Sciences, Chinese Academy of Sciences, Shanghai, 200032 China

**Keywords:** Embryo callus, Epigenome, Maize, MeDIP_seq, 24 nt small RNAs

## Abstract

**Background:**

Maize (*Zea mays*) is an important model crop for transgenic studies. However, genetic transformation of maize requires embryonic calli derived from immature embryo, and the impact of utilizing tissue culture methods on the maize epigenome is poorly understood. Here, we generated whole-genome MeDIP-seq data examining DNA methylation in dedifferentiated and normal immature maize embryos.

**Results:**

We observed that most of the dedifferentiated embryos exhibited a methylation increase compared to normal embryos. Increased methylation at promoters was associated with down-regulated protein-coding gene expression; however, the correlation was not strong. Analysis of the callus and immature embryos indicated that the methylation increase was induced during induction of embryonic callus, suggesting phenotypic consequences may be caused by perturbations in genomic DNA methylation levels. The correlation between the 21-24nt small RNAs and DNA methylation regions were investigated but only a statistically significant correlation for 24nt small RNAs was observed.

**Conclusions:**

These data extend the significance of epigenetic changes during maize embryo callus formation, and the methylation changes might explain some of the observed embryonic callus variation in callus formation.

**Electronic supplementary material:**

The online version of this article (doi:10.1186/s12870-017-1055-x) contains supplementary material, which is available to authorized users.

## Background

Maize is one of the most important crops for both human and livestock animals. For several decades, maize has been modified using both conventional and molecular breeding methods to generate plants with an increased yield and a greater ability to adapt to various disadvantageous conditions. Efforts are also underway to create maize plants with improved yield traits and resistance to various stresses using genetic engineering techniques.

Genetically modified maize plants are usually generated via tissue culture, and maize has been a primary target for genetic manipulation. To date, genetic transformation of maize still largely depends on immature maize embryo-derived calli [1]. Genetically, maize is a diverse species [2, 3] with a complex genome encoding repetitive regions [4, 5]. However, methylation changes occur and are an important source of tissue culture-induced variation, which appears to be much more frequent than genetic sequence variation [6] and suggests that epigenetic mechanisms play a critical role in the cellular transformation and, ultimately, cellular phenotypes. There is evidence that epigenetic alternations in both plants and animals can lead to phenotypic variations [7–11]. However, the role of epigenetic variation, in particular during maize embryo callus induction, has not been well characterized.

Generally, plant genomic DNA is methylated in three cytosine contexts: CG, CHG, and CHH (H = A, T, or C). Previous studies have indicated that distinct genetic pathways participate in distinct methytransferase-regulated DNA methylation in these contexts in *Arabidopsis* [12]. However, the majority of genome-wide methylation studies were performed in *Arabidopsis* and in different maize lines and tissues [13–21]. In these studies, DNA methylation was closely associated with transposable elements and repetitive DNA. In general, methylation of promoter regions is correlated with gene expression, whereas methylation changes to gene body regions show low/no correlation with gene expression [14, 17]. More interestingly, little evidence reports consistent changes to maize DNA methylation patterns in response to specific and distinct stress treatments [21]. To some extent, the maize embryo callus can be induced under certain stress-like conditions such as induction by auxin/cytokinin or wounding, and although the induction conditions are different from specific stress treatments, we suspect that DNA methylation patterns change during maize embryo callus formation.

Therefore, we investigated the effect of callus initiation through dedifferentiation on the methylome of maize embryos. We generated genome-wide DNA methylation maps using methylated DNA immunoprecipitation sequencing (MeDIP-seq) in dedifferentiated maize embryos after callus induction and in normal immature maize embryos without induction. We observed that tissue culture of the embryos induced changes to DNA methylation. In most cases, we observed an increase in DNA methylation throughout the genome that was associated with small RNA expression (specially 24 nt small RNA), and these methylation changes were enriched at promoter regions. Elevated DNA methylation at promoters in dedifferentiated embryos was associated with alterations in the expression levels of particular genes.

## Methods

### Plant materials

Maize inbred line18-599R, a cultivar with high dedifferentiation capacity, was used in this study. It was cultivated and is currently kept by Maize Research Institute of Sichuan Agricultural University. For DNA preparation in this study, all inbred line 18-599R seedlings, previously described in [22], were grown in the growth chamber at 27 °C with humidity of 70%. In brief, after 12 days (d) of self-pollination, immature ear of each plant was harvested. The immature embryos (1.5 mm–1.8 mm) were isolated and cultured with optimized N6 medium aseptically at 27 °C in darkness for 15 d. Generally, after inoculating, the immature embryos were divided into three stages according to their morphological features [22]: 1–5 d (intumescent embryo, Stage I), 6–10 d (initial callus formation, Stage II), and 11–15 d (embryonic callus formation, Stage III). Samples were collected from three individuals each day and pooled for three biological replicates at each stage. The embryos from immediately harvested ears without inoculation were collected with three biological replicates and used as a control group (0 d, CK) in this study.

### DNA extraction and methylated DNA immunoprecipitation sequencing (MeDIP-seq)

Genomic DNA was extracted from the samples using TaKaRa Universal Genomic DNA Extraction Kit Ver. 3.0 (DV811A) (TaKaRa, Osaka, Japan) according to the manufacturer’s instructions. In total, 12 genomic DNA samples (three biological replicates at each of the four stages) were sonicated to produce DNA fragments ranging from 100 bp to 500 bp. After DNA end-repair and 3’dA-tailing using the Paired-end DNA Sample Prep Kit (Illumina, San Diego, CA, USA), the DNA samples were ligated to Illumina sequencing primer adaptors. Double-stranded DNA was denatured and immunoprecipitated using an anti-5-methycytosine monoclonal antibody (Zymo Research, Orange, CA, USA). For each sample, the following procedures were performed as described [23]. 220 bp to 320 bp bands were excised and purified from the immunoprecipitation gel and quantified using an Agilent 2100 Analyzer (Agilent Technologies, Santa Clara, CA, USA). Finally, ultra-high-throughput 50 bp paired-end sequencing was performed using the Illumina HiSeq 2000 (BGI, Shenzhen, China) according to the manufacturer’s protocols.

Paired-ended sequencing raw reads (PE 50 bp) generated from MeDIP-seq were used to remove the containing adaptors and low quality reads with default settings. The clean reads (remaining reads) were aligned to the maize genome (RefGen_v3) [5] using Soap2 [24], allowing up to 2 bp mismatches to the reference genome and only returning uniquely mapped reads. MeDIP-seq data were analyzed using the R/Bioconductor package MEDIPS [25]. For each sample, the aligned reads were extended to a length of 300 bp in the sequencing direction. The genome was divided into adjacent 500 bp windows, and all additional calculations were applied to each window. Subsequently, methylation levels were quantified using MEDIPS to produce the relative methylation signal values (RMS) for further analysis. The mean relative methylation score (RPM) in each window across various regions of interest (e.g., promoters, 5′-UTR, 3′-UTR, exons, introns, CpG islands (CGIs)) was used to analyze the differentially methylated regions (DMRs).

### DMRs discovery and annotation

For DMRs estimation, the RPM values in the control group (0 d, CK) were compared to each inoculated group stage I, II, and III. Differentially methylated regions were identified by applying edgeR for testing windows across regions of interest distributed throughout the genome. Significance of the results form DMRs analyses was estimated with *P*-value <0.05. Each DMR was annotated using *Zea mays* genes (AGPv3 (5b)) (http://plants.ensembl.org/biomart/martview/242ebdeebecbf2ed59df0f7230470954).

### Digital gene expression (DGE) profiling data analysis

DGE data were utilized from [22], and were reanalyzed in this study. Generally, the DGE data were processed using SOAP2 with default parameters. After removing adapters and low-quality reads, the clean tags were mapped to the maize reference genome v3 (RefGen_v3), then the uniquely mapped tags were normalized to TPM (number of transcripts per million clean tags), and used to analyze differentially expressed genes (DEGs) using edgeR [26]. The DEG results were estimated with a combination of FDR < 0.001 and the absolute value of log2-Ratio ≥ 1. For further methylation analysis, all genes from DGE profiling mentioned below were differentially expressed genes. The analysis was followed that of Regulski et al. [27]*.*

### Small RNA-seq data analysis and calculation of methylation in TEs

The small RNA-seq data were utilized from [28] and reanalyzed in this study. Generally, data were filtered with SOAP2 using default parameters. The clean small RNA reads were mapped to the maize reference genome v3 (RefGen_v3) with a maximum of 2 mismatches. To estimate correlations between small RNA and methylation profiles in 2 kb upstream regions, the normalized read counts for small RNAs were used for calculations.

Reads mapped to transposable elements (TEs) were normalized as previously described [27].

### Data access

The data from this study have been deposited in the NCBI Gene Expression Omnibus (GEO; http://www.ncbi.nlm.nih.gov/geo/) and are accessible through GEO Series accession number GSE84455.

## Results

### MeDIP-seq analysis of dedifferentiation in maize embryo reveals a large number of differentially methylated regions

**Fig. 1:**
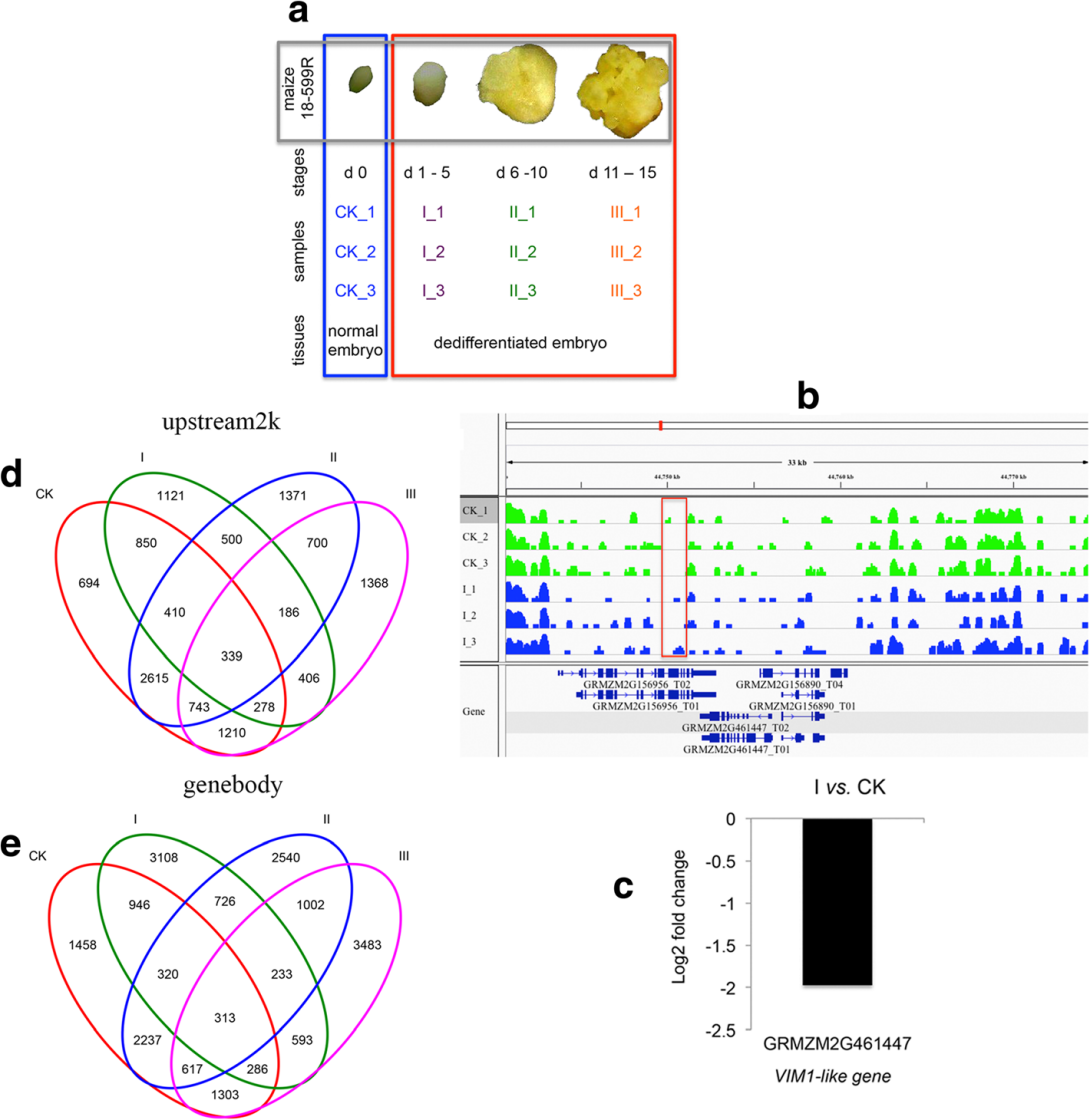
Generation of genome-wide methylation maps for immature embryos and callui. **a** Summary of samples used for genome-wide methylation analyses. Normal embryo tissues (CK) and dedifferentiated embryo samples (I, II, and III) were employed for MeDIP-seq, DGE, and small RNA-seq. **b** Visualization of hypermethylated DMRs in the CK group and stage I-callus within the *VIM1-like* gene (GRMZM2G461447) using the IGV tool. *Green*, *blue*: MeDIP-seq tracks of the CK group and each embryo callus stage, respectively; *red outlines* the hypermethylated region of the gene of interest. **c** Expression of the *VIM1-like* gene, as determined by DGE. The expression is given as the log2-fold change calculated comparing the stage I-callus to the CK group (normal embryos). **d**–**e** Venn diagram showing the DMRs identified in 2 kb upstream and gene body regions

To investigate possible DNA methylation patterns changes that occur during callus induction in the maize embryo, we compared the methylated DNA of normal and inoculated embryos from the maize inbred line 18-599R using immunoprecipitation followed by massively parallel sequencing (MeDIP-seq). Samples were immediately collected 12 d after self-pollination, and inoculated embryos were collected at each stage (Fig. 1a; immature embryos without inoculation (CK), intumescent embryo (stage I), initial calli (stage II), and embryonic calli (stage III)) and were assessed with MeDIP-seq to generate a total of approximately 1.16 × 10^9^ reads (average length 50 bp). The chromosomal distribution of DNA methylation reads for each maize embryo sample is depicted in Additional file 1: Fig. S1. In general, an average of 92.85% reads of the total reads aligned to the maize B73 reference genome, of which approximately 37.55% reads were uniquely mapped (3.64 × 10^7^ reads; see Additional file 2: Table S1 for mapping statistics). To test for correlations between the MeDIP-seq samples, we calculated the Pearson’s correlation coefficients based on read counts of the uniquely mapped reads. The results revealed a moderate to high overall similarity between samples (*r* = 0.56–0.92; Additional file 2: Table S2). The pairwise correlations between MeDIP-samples derived from the same dedifferentiated stage were mostly above 0.80. In contrast, the pairwise correlations between the CK group and each other stage were mostly below 0.80, indicating a difference in global methylation after treatment.

To identify differentially methylated regions (DMRs) between the CK group and the other stages, we calculated and compared the read density in overlapping 500 bp windows across the maize genome (described in the Methods section; *P* < 0.05; mean signal in at least one group > 0.25 reads per million; |ratio between CK and the other stage| > 2). We identified 7036 differentially methylated regions (DMRs, size range 500 bp), of which 5376 (76.41%) were hypermethylated and 1660 (23.59%) were hypomethylated when comparing between stage I and the CK group (For example, see Fig. 1b and c for a hyermethylated DMR in the promoter region of *VIM1-like gene* GRMZM2G461447). A total of 18,887 DMRs were identified in stage II (compared to CK), exhibiting 12,372 (65.51%) hypermethylated and 6515 (34.49%) hypomethylated regions; 11,514 DMRs were observed in stage III (compared to CK) with 9773 (84.88%) hypermethylated and 1741 (15.12%) hypomethylated regions (Table 1; see Additional file 2: Table S3 for full list of DMRs across different comparisons). Among these DMRs, 339 and 313 were consistently detected across all of the stages in the promoter region (Fig. 1d, upstream flanking 2000 bp region) and gene body regions (Fig. 1e), respectively. Moreover, 694, 1121, 1371, and 1368 DMRs were uniquely present in promoter regions from CK, stage I, II, and III samples, respectively (Fig. 1d), whereas 1458, 3108, 2540, and 3483 DMRs uniquely appeared in the gene body regions from CK, stage I, II, and III embryos, respectively (Fig. 1e). Interestingly, we found 186 and 233 DMRs were consistent between all of the analyzed stages of callus induction in the promoter region and genebody region (Fig. 1d and e), respectively. Among these consistent DMRs, some may play important roles in the epigenetic manipulation due to the specificity to callus induction, such as dehydration-responsive element-binding protein 1B (*DgDREB1B*, GRMZM2G325513), which played an important role in plant development [29]; and 3-methylcrotonyl-CoA carboxylase (*MCCas*e, GRMZM2G702490), a nuclear-encoded as well as mitochondrial biotin-containing enzyme, which has been reported the physiological roles in maintaining the carbon status of organism [30].

**Table 1:** Numbers of DMRs identified by MeDIP-seq, and assigned to subgenomic regions^a^

Comp.	DMR	Total	CGI	Promoter	Exon	Intron	TTR
I vs. CK	Total	7036	830	2317	1684	1788	778
	Hyper	5376	525	1830	1375	1254	664
	Hypo	1660	305	487	309	534	114
II vs. CK	Total	18,887	3293	6728	4440	4006	2040
	Hyper	12,372	1213	4538	3315	2485	1696
	Hypo	6515	2080	2190	1125	1521	344
III vs. CK	Total	11,514	1419	3670	2899	2481	1376
	Hyper	9773	1108	3142	2544	1963	1273
	Hypo	1741	311	528	355	518	103
II vs. I	Total	5106	842	1413	1077	1440	432
	Hyper	2234	143	731	568	628	237
	Hypo	2872	699	682	509	812	195
III vs. II	Total	5765	1174	1643	1124	1595	453
	Hyper	3667	1074	923	604	942	237
	Hypo	2098	100	720	520	653	216
III vs. I	Total	3742	363	969	874	1055	358
	Hyper	2339	244	575	545	633	234
	Hypo	1403	119	394	329	422	124

^a^DMR, differentially methylated region; CGI, CG island; TTR, transcription termination region

### Ontology-based enrichment analysis identified biological processes related to differential promoter methylation in embryonic callus formation

**Fig. 2:**
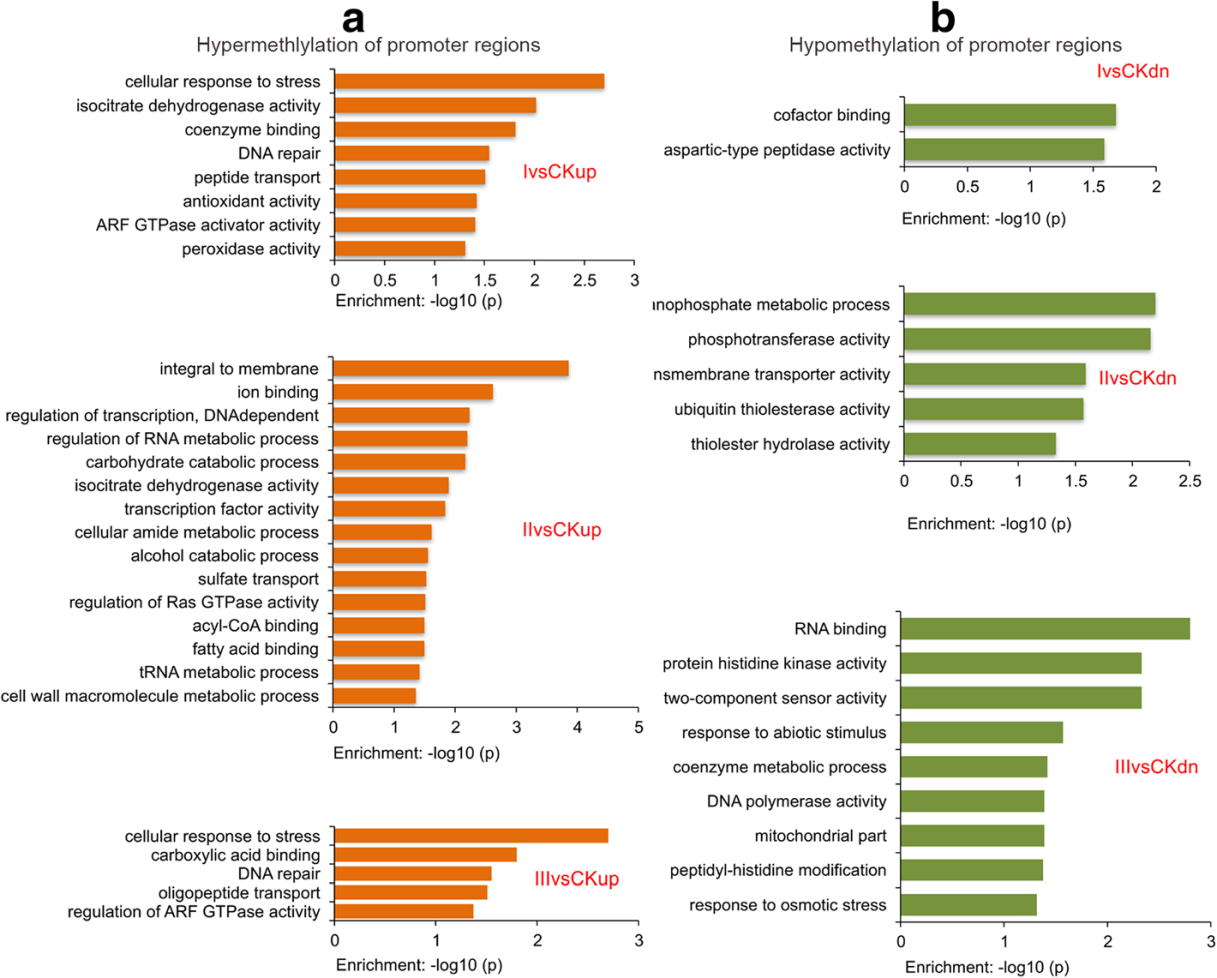
Molecular features of genes with differentially methylated promoter regions in embryo calli. The genes with hypermethylated (**a**) or (**b**) hypomethylated promoter regions were analyzed by gene ontology, and the significantly enriched (*P* < 0.05) GO terms are plotted

The presence of DNA methylation is often considered to result in lower level of transcription. However, genome-wide profiles of DNA methylation and gene expression have suggested that DNA methylation does not cause decrease of gene expression during the functional stages [10, 15]. Because we observed DMR enrichment in promoters (Additional file 2: Table S3), we performed gene ontology (GO) analysis on genes showing different promoter methylation using the Database for Annotation, Visualization and Integrated Discovery (DAVID) online tool (http://david.abcc.ncifcrf.gov/) to study the functional consequence of promoter methylation in an unbiased fashion [31]. Selected DAVID results are presented in Fig. 2, while all results are presented in Additional file 2: Table S4 (*P* < 0.05). Interestingly, for the hyper-methylated regions, the GO terms over-represented in comparisons Ivs.CKup and IIIvs.CKup analysis (e.g. cellular response to stress) seems more similar to each other than that in IIvs.CKup (e.g. regulation of transcription, DNA dependent). The function annotated from these comparisons by DAVID is consistent with the biological process of embryonic calli formation.

We found that the most enriched functional categories in the hypermethylation group were related to cellular responses to stress, DNA repair, DNA-dependent regulation of transcription, and responses to DNA damage, among others (Fig. 2a). Interestingly, we identified ion binding to be a uniquely enriched functional category in stage II (initial callus), which suggests that a number of genes, perhaps specifically encoding enzymes, might be involved in this process. Meanwhile, the finding of functions related to ARFs is interesting. In total, four genes (GRMZM2G176495, GRMZM2G126079, GRMZM2G054821, GRMZM2G083546) were observed to be enriched in the regulation of ARF protein signal transduction. We also performed GO analysis on the hypomethylation group and ranked the enriched GO terms according to their *p*-value (Fig. 2b). The top enriched terms were relevant to RNA binding, phosphotransferase activity, and co-factor binding. This indicates that the phosphorylation of several factors including blue-light receptor phototropin 1 (phot1, GRMZM2G001457), blue-light receptor phototropin 2 (phot2, GRMZM2G032351), phytochromeC2 (phyC2, GRMZM2G129889), and histidine kinase1 (hk1, GRMZM2G151223), is severely affected by tissue culture conditions. These results imply that factors responding stresses (e.g. darkness, auxin) and initiated in a DNA-methylation manner (e.g. through protein phosphorylation) might indirectly contribute to embryonic callus growth.

### Differential promoter methylation and differential gene transcription in embryonic calluses are not highly correlated

**Fig. 3:**
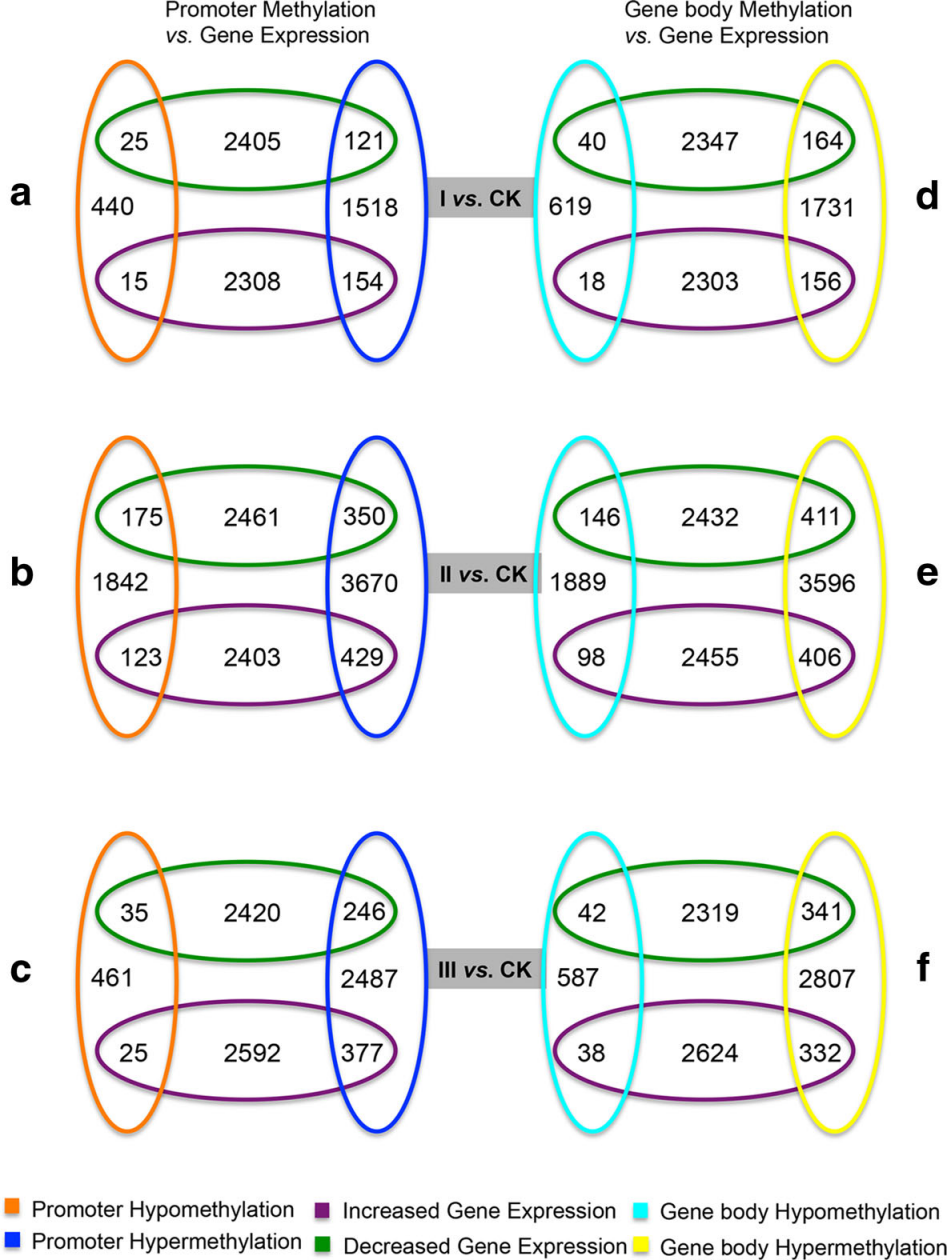
Differential gene methylation and differential gene transcription in embryo calli are not highly correlated. **a**–**f** Venn diagrams display numbers of differentially methylated and transcriptionally regulated genes. The cut-off criteria are <0.05 for methylation (calculated using MEDIPS) and FDR < 0.001 for transcriptional regulation. **a**–**c** Genes with different promoter methylation in the embryo callus compared to the CK group. **d**–**f** Genes that display differential gene body methylation during embryo callus formation

**Fig. 4:**
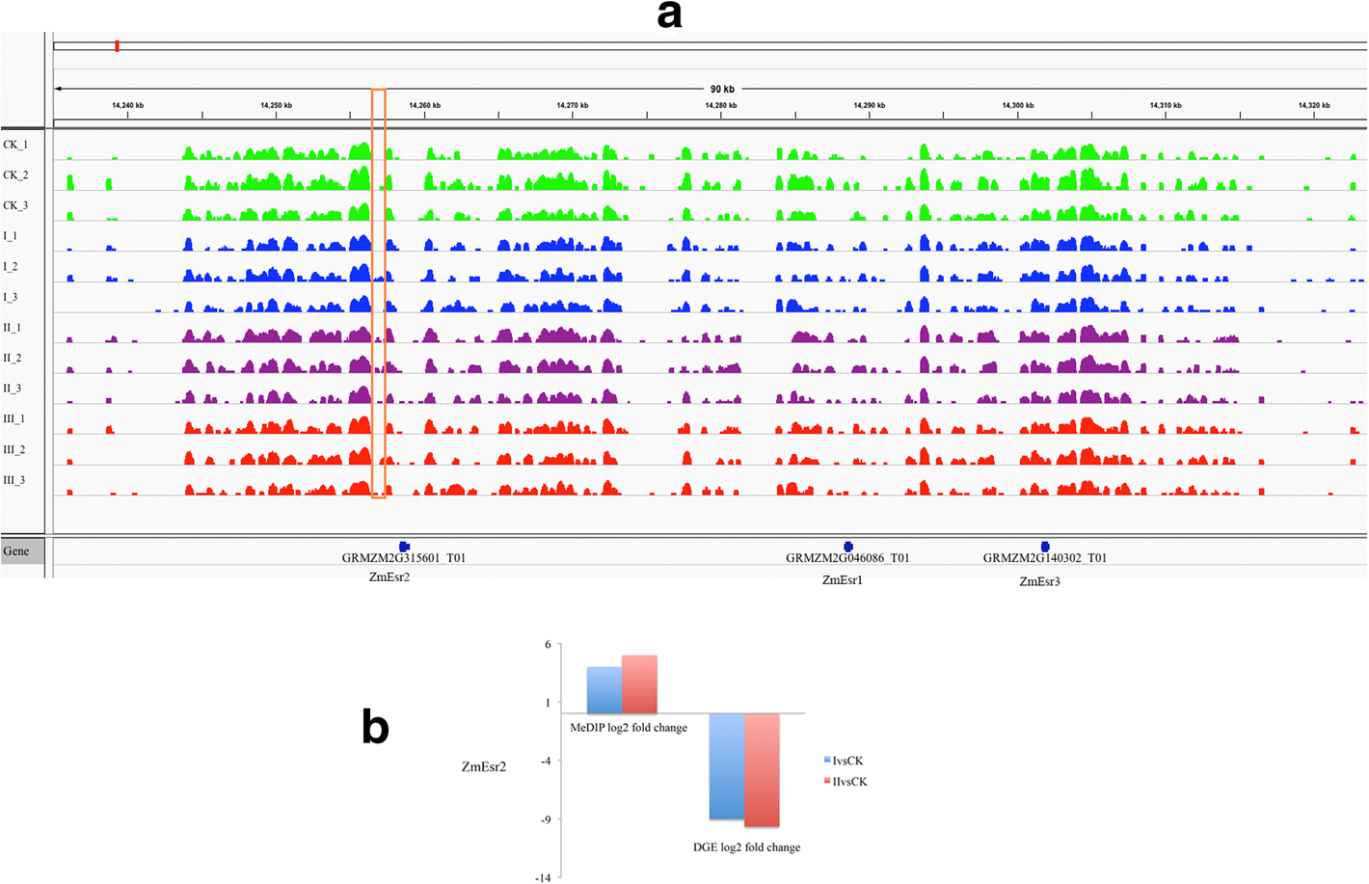
Association of hypermethylation with transcriptional down-regulation at the ZmEsr2 locus. **a** Track of the MeDIP-seq data using the IGV tool. *Green*, *blue*,*purple*, and *red*: MeDIP-seq tracks for the CK group and each embryo callus stage; *red outlines* the hypermethylated region of the gene of interest. **b** Expression of *ZmEsr2* and the neighboring *ZmEsr1* and *ZmEsr2* genes as determined by DGE. Expression is provided as the log2-fold change. ZmEsr2 was significantly down-regulated (FDR < 0.001)

**Fig. 5:**
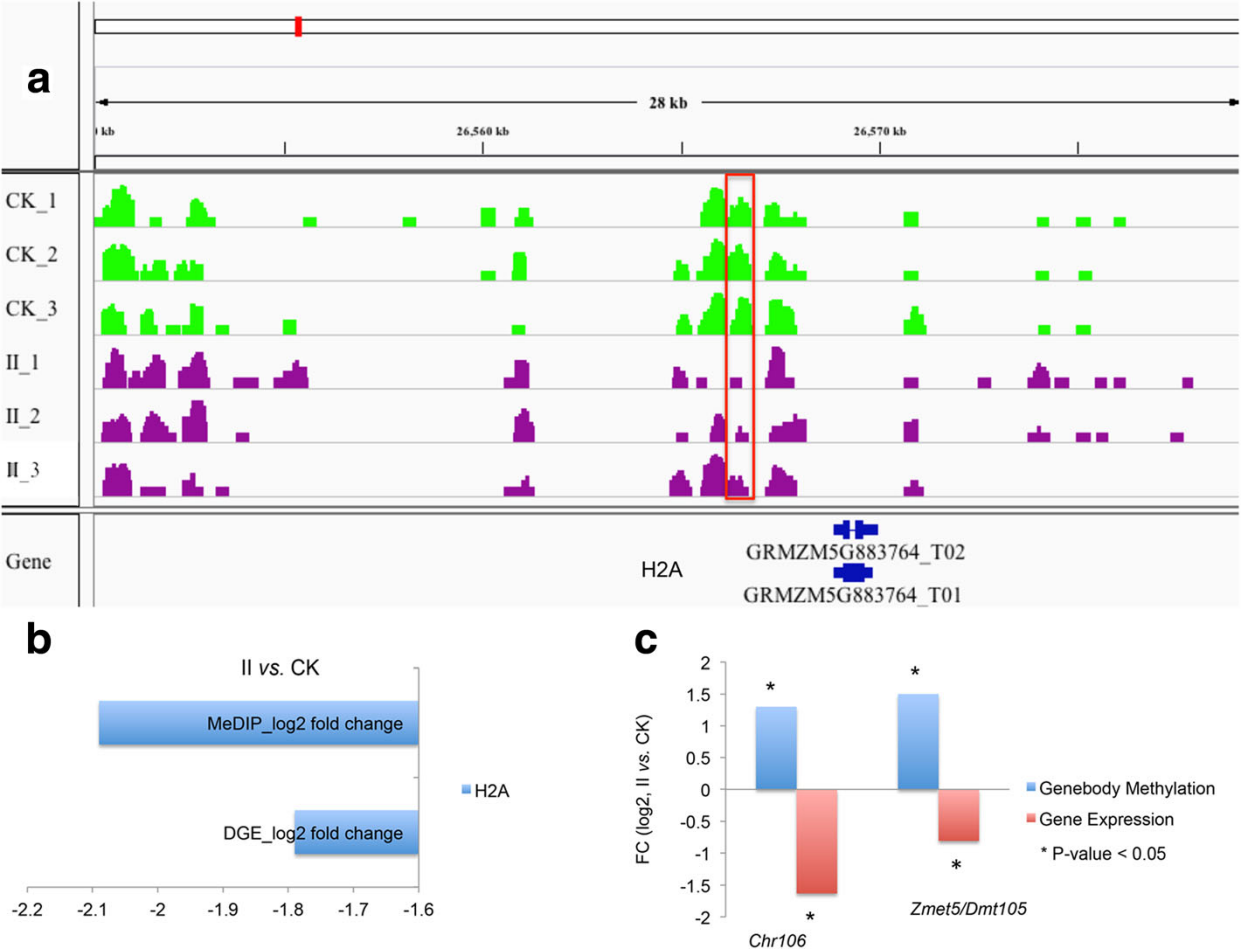
Association of methylation and transcriptional down-regulation at a different locus. **a** IGV track of the MeDIP data at the *H2A* locus. *Green*, *purple*: MeDIP-seq tracks of the CK and stage II embryo callus groups, respectively; *red outlines* the hypomethylated region of the gene of interest. **b** H2A methylation and expression as determined by DGE. Expression is given as the log2-fold change as calculated for embryo callus compared to normal embryo. **c** Association of hypermethylation and down-regulation at the *chr106* and *Zmet5/ Dmt105* loci. Both genes are significantly down-regulated (FDR < 0.001)

It is generally assumed that promoter hypermethylation is correlated with down-regulation of the gene, whereas promoter hypomethylation is correlated with up-regulation [14, 17]. However, this might not be true during maize embryonic callus development because a previous study provided little evidence to support consistent changes in maize DNA methylation patterns in response to performing different specific stress treatments [21]. To understand the effect of hypermethylation or hypomethylation on gene expression, we reanalyzed high throughput RNA-sequencing data [22] on the same stages of tissue samples that were used for MeDIP-seq (see Additional file 1: Fig. S2 and Additional file 2: Table S5 for digital gene expression (DGE) data assessment and Additional file 2: Table S6 for list of differentially expressed genes). Generally, 1544 and 1523 genes were up-regulated (Additional file 1: Fig. S2A) and down-regulated (Additional file 1: Fig. S2B) in all stages of embryonic callus formation. The Kyoto Encyclopedia of Genes and Genomes (KEGG) pathway analyses resulted from DAVID online tool identified significantly over-represented pathways relation to starch and sucrose metabolism, carbon fixation in photosynthetic organisms (Additional file 1: Fig. S2C) in the up-regulated genes and DNA replication, Citrate cycle (TCA cycle) in the down-regulated genes (Additional file 1: Fig. S2D), respectively. We evaluated the genes that were, both differentially methylated and differentially expressed between the CK group and each embryonic callus stage (I, II, and III). The genes that were hypermethylated at their promoters and down-regulated during callus induction has different numbers (Fig. 3a–c; 121 genes in stage I, 350 in stage II, and 246 in stage III). One example is the *ZmEsr2* gene (CLAVATA3/ESR (CLE)-related protein 2-B ESR2Bp, GRMZM2G315601). Promoter hypermethylation is correlated with downregulation of the *ZmEsr2* gene (Fig. 4), which is a known cytokinin-signaling molecule involved in developmental processes during maize embryo development [32, 33]. Likewise, promoter hypomethylation correlated with increased gene expression for several genes (15 genes in stage I, 123 in stage II, and 25 in stage III), but the overlap between genes with hypomethylated promoters and transcriptionally up-regulated genes was less extensive (Fig. 3a–c). However, some of the genes displayed a similar pattern between promoter hyper-methylation and down-transcriptional activity, or between hypo-methylation and up-transcriptional activity, although some genes showed an inverse pattern. For example, *H2A* (Histone H2A, GRMZM5G883764) contained a hypomethylated DMR in its promoter in stage II (compared to the CK group, Fig. 5a); however, this did not increase expression at stage II (Fig. 5b), although the gene plays an important role in dedifferentiated callus [34]. We also evaluated genes that were differentially expressed in the callus and that show changes to gene body methylation, although only a small overlap was observed between gene body methylation and gene expression (Fig. 3d–f).

### DNA hypermethylation in embryo calli occurs at genes that might influence DNA methylation patterns in maize

**Fig. 6:**
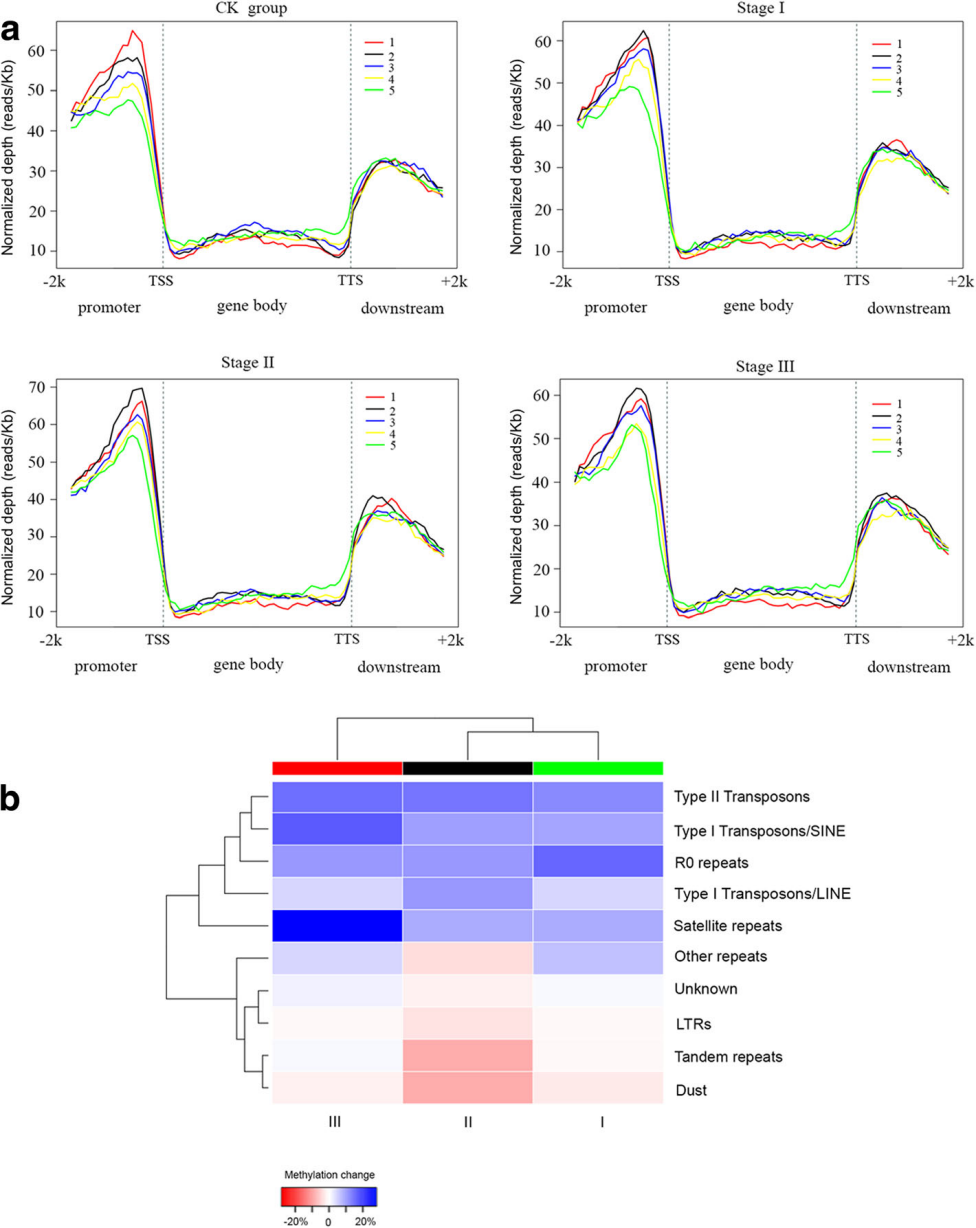
Distribution of DNA methylation patterns in genes and TEs with different expression levels. **a** Gene expression levels (RPKM values) calculated from DGE data were classified into five categories, where “1” indicates the highest expression level and “5” indicates the lowest expression level. The y-axis represents normalized depth (reads/Kb). **b** Methylation change was calculated (as stage I, II or III - CK)/CK, and the values for each stage and the CK group are the average of the three replicates. The color *red*, *black*, *green* represent stage I, II, and III, respectively

Previous data revealed that collections of mutant alleles for 11 maize genes were predicted to play roles in DNA methylation [35]. We thus assessed the promoter/gene body methylation and transcriptional activity of these genes potentially involved in maize DNA methylation. In the maize embryo-derived callus, however, none of these 11 genes were both promoter-hypermethylated and transcriptionally silenced, although the whole-genome methylation pattern showed greater hypermethylation in promoter regions compared to gene body regions (Fig. 6a). However, two out of the 11 genes (*Chr106*, GRMZM2G071025; *Zmet5/Dmt105*, GRMZM2G005310) were both genebody-hypermethylated and transcriptionally down-regulated at stage II (Fig. 5c) and were not differentially expressed at other stages (compared to CK). *Zmet5/Dmt105* is a full-length chromomethylase gene in maize genome that is closely related to *Arabidopsis CMT3*, which is an important methytransferase [35]. *Chr106* is similar to *Arabidopsis DDM1* and function as a chromatin remodeler.

Interestingly, the *mediator of paramutation 3* (*mop3,* GRMZM2G007681) was hypermethylated in both the promoter and genebody regions during stage I (compared to CK) and hypermethylated in genebody regions in stage III (compared to CK); however, no *mop3* transcriptional changes were observed during these two stages (Additional file 2: Table S3). Instead, the *mop3* mRNA level was up-regulated in stage II (compared to CK) (Additional file 2: Table S6), although we did not find any DMRs of this gene at stage II (compared to CK).

### Changes in DNA methylation levels at transposable elements differ after callus induction

Transposable elements (TEs), which were first discovered in maize, are abundant and dynamic and play important roles in the evolution of genes and genomes in multiple organisms [36]. Previous studies found that methylation is guided by small RNAs and is correlated with transposon insertion [27]. We therefore asked whether the methylation signature of TEs were different; for instance, whether small RNAs guide methylation patterns during embryo callus formation (Fig. 6b). To this end, we identified that both type I and II TEs displayed hyper/hypo-methylation patterns during embryo callus formation. For type II transposons and type I transposons/SINE, extensive hypermethylation changes were observed at each embryo callus stage compared to CK, whereas hypermethylation of type I transposons/LINE only occurred at stage II (Fig. 6b). Type II TEs transpose by mobilizing DNA directly via a cut-and-paste mechanism, whereas type I TEs transpose by reverse transcription of a transcribed RNA [36, 37]. Other type I TEs,the major class of TEs called long terminal repeats (LTRs) retrotransposons [38], showed broad hypomethylation changes during each stage,with stronger hypomethylation at stage II (initial callus). Several studies demonstrated that type I elements, especially LTRs, contribute primarily to the dynamic gene function and evolution in higher plants. Some LTRs might amplify gene fragments and occasionally fuse to genes to create novel genetic functions [36, 39], leading to chromosomal rearrangements such as deletions, duplications, and translocations. Therefore, we further identified LTR subtypes as well as the other type I/II TEs using the available maize transposable element database (http://maizetedb.org/~maize/) (Additional file 2: Table S7). Strikingly, we found that the majority of methylation level changes to TEs were at LTRs (see subtypes in Additional file 2: Table S7), suggesting potential roles for LTRs in embryonic callus formation.

Finally, we compared the levels of methylation with matching small RNAs [28] isolated from the same tissues as described in the Methods section. Small RNA data [28] generated from the same tissues used for MeDIP-seq (Additional file 2: Table S1) were mapped to the maize B73 genome (v3) and the transposable element database using Bowtie as previously described [27, 40]. Table 2 presents the correlations between 21, 22, 24-nt small RNAs and methylation. As shown in the table, the methylation level was not strongly correlated with 21-nt and 22-nt small RNAs levels. However, similar to a previous study [27], 24-nt small RNAs was significantly positively correlated with DNA methylation at each analyzed stage of callus induction but was negatively correlated with methylation in the CK group (*P* < 0.05, Table 2). To describe the targets of the 24-nt small RNAs and to further describe the potential changes in expression in the pathways, we used a plant small RNA target analysis server (psRNATarget) [41] to map the target genes. All targets of the 24-nt small RNAs at each stage were listed in Additional file 2: Table S8. Finally, we identified 566 genes that are consistently targeted by 24-nt small RNAs among all the stages (stage I, II, and III, Additional file 1: Fig. S3). A previous study reported that the 24 nt small RNAs are associated with RNA-dependent DNA methylation (RdDM) that may give rise to transcriptional gene silencing. Furthermore, a study on the root meristems of *Arabidopsis thaliana* indicated the significance of (24-nt) RNA silencing signal to embrace epigenetics and transcriptional gene silencing [42]. Intriguingly, pathway analysis of the identified 566 target genes results from DAVID indicates that the pathway zma03040: Spliceosome (http://www.genome.jp/kegg-bin/show_pathway?map03040) was over-represented, which involved five target genes (GRMZM2G020728, GRMZM2G171372, GRMZM2G003307, GRMZM2G100620, GRMZM2G031827). One of the players in the spliceosome pathway, splicing factor U2AF subunit (GRMZM2G031827), was found to be targeted by 24-nt small RNA (UAGGUUAUUCCUUUUGGUGUAGGC) and play a very important role in RNA splicing, indicates a potential novel signal where they caused epigenetic changes that may influence induction and development of maize embryo callus.

**Table 2:** Small RNA guided methylation^a^

Length of small RNAs	CK		I		II		III	
	corrlation	*p*-value	corrlation	*p*-value	corrlation	*p*-value	corrlation	*p*-value
21	0.1404	0.5664	0.2407	0.3692	0.1815	0.4587	−0.0194	0.941
22	−0.2584	0.2569	0.1956	0.3382	0.3652	0.0548	0.4434	0.016
24	−0.4285	0.0065	0.4821	0.0012	0.4522	0.0005	0.5309	0.0001

^a^Correlation coefficents, calculated as described [26]

## Discussion

For the first time, we compared methylated DNA from primary normal immature maize embryo to dedifferentiated cultures from the same organ using immunoprecipitation followed by massively parallel sequencing (MeDIP-seq). We observed that the callus-specific DNA methylation patterns were distinct from those found in normal immature embryos. These data indicate that callus-specific DMRs do not pre-exist in the cell population as a minor component of the maize embryo that emerge by expansion of the embryo callus cell type. These experiments establish that epigenetic patterns observed in dedifferentiated maize embryo cultures result from callus induction and will thus contribute to specific epigenetic manipulation.

Hypermethylation events were observed more frequently than hypomethylation events following callus initiation and formation during maize embryo dedifferentiation, which differs from embryonic callus formation for plant regeneration (re-differentiation process) but can ultimately be reflected in phenotypical variability of regenerated maize plants as described [43]. In our study, we mainly focused on the dedifferentiation process, which is characterized by more hypermethylation events. This might prepare the plant for later regeneration with increased hypomethylation, which is consistent with a previous study [43]. Stelpflug et al. [43] reported that decreased DNA methylation following tissue culture was more common than increase of DNA methylation during plant regeneration. For instance, indole-3-acetate beta-glucosyltransferase (GRMZM5G896260) was observed as hypermethylated DMR in the promoter region at stage III compared to the CK group, consistently, GRMZM5G896260 was detected as hypomethylated DMR (DMR ID 354) in the regenerated plant as described [43].

Generally, current epigenomic models assume that DNA hypermethylation, especially promoter methylation, is a negatively correlated with gene expression [17]and indicates gene silencing. We found that with respect to maize embryo calli, this promoter-model is only accurate for a minority of genes with hypermethylated promoters (Fig. 3a–c). Likewise, only a minor fraction of genes with hypomethylated promoters are transcriptionally up-regulated in embryo callus (Fig. 3a–c). These groups of genes occur more frequently in embryo calli than expected by chance; however, the large majority of detected genes do not follow conventional rules. Overall, changes in promoter methylation do not appear to significantly alter gene expression. Additional research is required to futher elucidate the regulation of gene expression by epigenetic mechanisms involving additional control elements such as enhancers and intragenic silencers in maize embryo calli.

Previous studies found that regions of DNA methylation within gene bodies were widely observed to have little to no influence on gene expression [15, 44, 45], whereas DNA methylation in the first hundred base pairs of a gene is associated with changes to gene expression [46]. Although the exact role of gene body methylation remains unclear, it might moderately influence transcribed genes [14, 17]. However, we find the gene body model to be consistent with the rules as previously described [14, 17]. A larger fraction of genes with genebody hypermethylation show changes in gene expression, whereas hypomethylation of the gene body leads to smaller changes in gene expression (Fig. 3d–f). This is an interesting phenomenon ignored by previous studies that should be thoroughly investigated in the future research on the maize epigenome, particularly in maize embryo dedifferentiation studies.

Although little to no correlation was observed between genebody methylation and gene expression, Regulski et al. [27] found that genebody methylation might prevent transposon insertion, disrupting gene function. Interestingly, Eichten et al. reported that genes located near retrotransposons were expressed at significantly lower levels in all of the examined maize genotypes and tissues [18], and DNA methylation differences associated with local genetic variation were observed near TEs [47]. In this study, we found substantial changes in methylation levels at transposable elements, most of which occurred at type I TEs/LTRs (Fig. 6) that are associated with chromosomal rearrangements such as deletions, duplications, and translocations [36, 39], which is consistent with previous reports [46].

## Conclusions

In summary, our data define a core methylation signature of maize embryo dedifferentiation, which is of great importance for genetic manipulation. The comparison of immature embryo-derived callus with normal immature embryo indicated that this core signature is established early during embryonic callus formation and is retained when the embryonic callus epigenome is modified during embryo intumescence progression to embryonic callus.

## Additional files

Additional file 1: Fig. S1. Chromosomal distribution of DNA methylation read for each maize embryo sample. Each chromosomal was split in 10Kb windows. **Fig. S2.** Comparative and pathway analysis of DGE data. (A, B) Venn diagrams display the intersection of differentially expressed genes as determined by FDR < 0.001 and log2fold change >1 for genes A) up-regulated and B) downregulated in differentiated embryo compared to normal embryo (CK group) (I vs. CK, II vs. CK, III vs. CK). C, D) KEGG pathway analyses. Overrepresented KEGG pathways in genes up-regulated (C) and down-regulated (D) in differentiated embryos compared to CK group as calculated (*P* < 0.05) are shown. The x-axis displays the –log10 of the *p*-values calculated by DAVID (http://david.abcc.ncifcrf.gov). **Fig. S3.** Venndiagram of 24-nt small RNA target DMRs and pathway results from DAVID. Venn diagrams display the intersection of target genes of 24-nt small RNAs that significantly positive correlated with DMRs. (PDF 1136 kb)

Additional file 2: Table S1. Sequencing statistics of MeDIP-seq, mRNA-seq, and small RNA-seq data. **Tabel S2.** Pairwise Pearson’s correlation coefficients (r) based on read counts of uniquely mapped reads. **Table S3.** Differentially methylated regions. **Table S4.** Data for Fig. 2. **Table S5.** Data for Additional file 1: Fig. S2. **Table S6.** DGE in stages vs. CK. **Table S7.** TE changes in stages vs.CK. **Table S8.** Data for Additional file 1: Fig. S3. (XLS 15783 kb)

## Abbreviations

CGI: CpG island
CK: Control group
DAVID: Database for Annotation, Visualization and Integrated Discovery
DEG: Differentially expressed genes
DGE: Digital gene expression
DMRs: Differentially methylated regions
GO: Gene ontology
KEGG: Kyoto Encyclopedia of Genes and Genomes
LTRs: Long terminal repeats
MeDIP-seq: Methylated DNA immunoprecipitation sequencing
RdDM: RNA-dependent DNA methylation
RMS: Relative methylation signal values
RPM: Mean relative methylation score
TE: Transposable element
TPM: Number of transcripts per million clean tags
TTR: Transcription termination region
