# Supplementary material for: Integrative analysis of DNA methylation, mRNAs, and small RNAs during maize embryo dedifferentiation
Source: BMC Plant Biol. 2017 Jun 15;17:105. doi: 10.1186/s12870-017-1055-x (PMC5472921; doi:10.1186/s12870-017-1055-x)

Integrative analysis of DNA methylation, mRNAs, and small RNAs during maize embryo dedifferentiation

Hongjun Liu[1,2+¶], Langlang Ma[1+], Xuerong Yang[2+], Lin Zhang[3], Xing Zeng[3], Shupeng Xie[4], Huanwei Peng[5], Shibin Gao[1], Haijian Lin[1], Guangtang Pan[1], Yongrui Wu[6], Yaou Shen[1]*

[1]Key Laboratory of Biology and Genetic Improvement of Maize in Southwest Region, Maize Research Institute, Sichuan Agricultural University, Chengdu, 611130, China.

[2]State Key Laboratory of Crop Biology, College of Life Sciences, Shandong Agricultural University, Tai'an, 271018, China.

[3]Department of Agronomy, Northeast Agricultural University, Harbin, 150030.

[4]Suihua Sub-academy, Heilongjiang Academy of Agricultural Sciences, Suihua, 152052, China.

[5]Institute of Animal Nutrition, Sichuan Agricultural University, Ya'an, 625014, China.

[6]National Key Laboratory of Plant Molecular Genetics, Institute of Plant Physiology & Ecology, Shanghai Institutes for Biological Sciences, Chinese Academy of Sciences, Shanghai 200032, China.

[+]these authors contributed equally to this work.

¶ Current address: State Key Laboratory of Crop Biology, College of Life Sciences, Shandong Agricultural University, Tai’an, 271018, China

[*] Corresponding author: Yaou Shen, shenyaou@gmail.com, 211 Huimin Rd., Wenjiang District, Chengdu, Sichuan, China. Tel: (+86) 028- 86290916

Running Title: DNA methylation in maize embryo dedifferentiation

Supplementary Materials

Figure S1. Chromosomal distribution of DNA methylation read for each maize embryo sample. Each chromosomal was split in 10Kb windows.

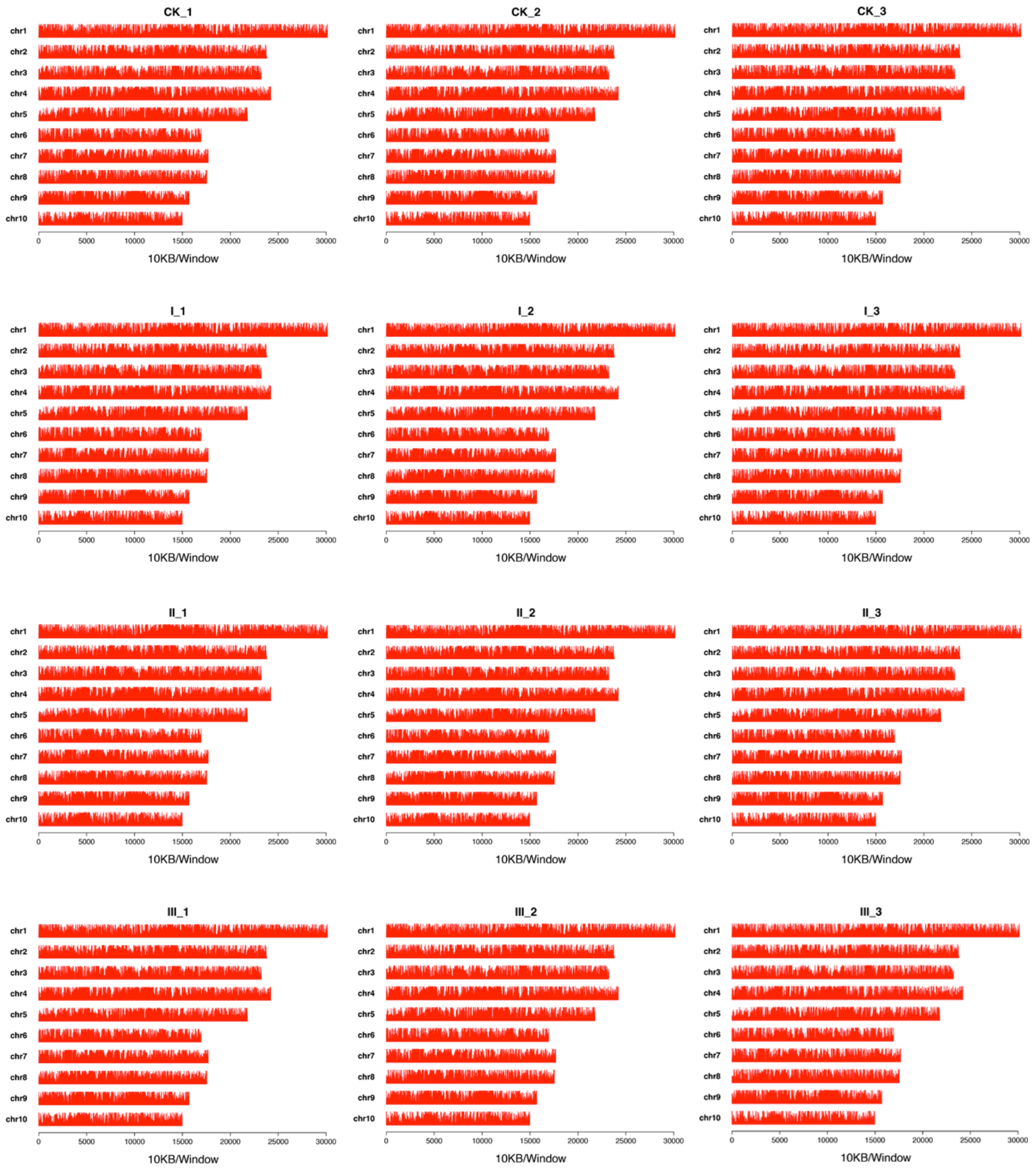

Figure S2. Comparative and pathway analysis of DGE data. (A, B) Venn diagrams display the intersection of differentially expressed genes as determined by FDR < 0.001 and log2fold change > 1 for genes A) up-regulated and B) down-regulated in differentiated embryo compared to normal embryo (CK group) (I *vs.* CK, II *vs.* CK, III *vs.* CK). C, D) KEGG pathway analyses. Overrepresented KEGG pathways in genes up-regulated (C) and down-regulated (D) in differentiated embryos compared to CK group as calculated (P < 0.05) are shown. The x-axis displays the –log10 of the p-values calculated by DAVID (http://david.abcc.ncifcrf.gov).

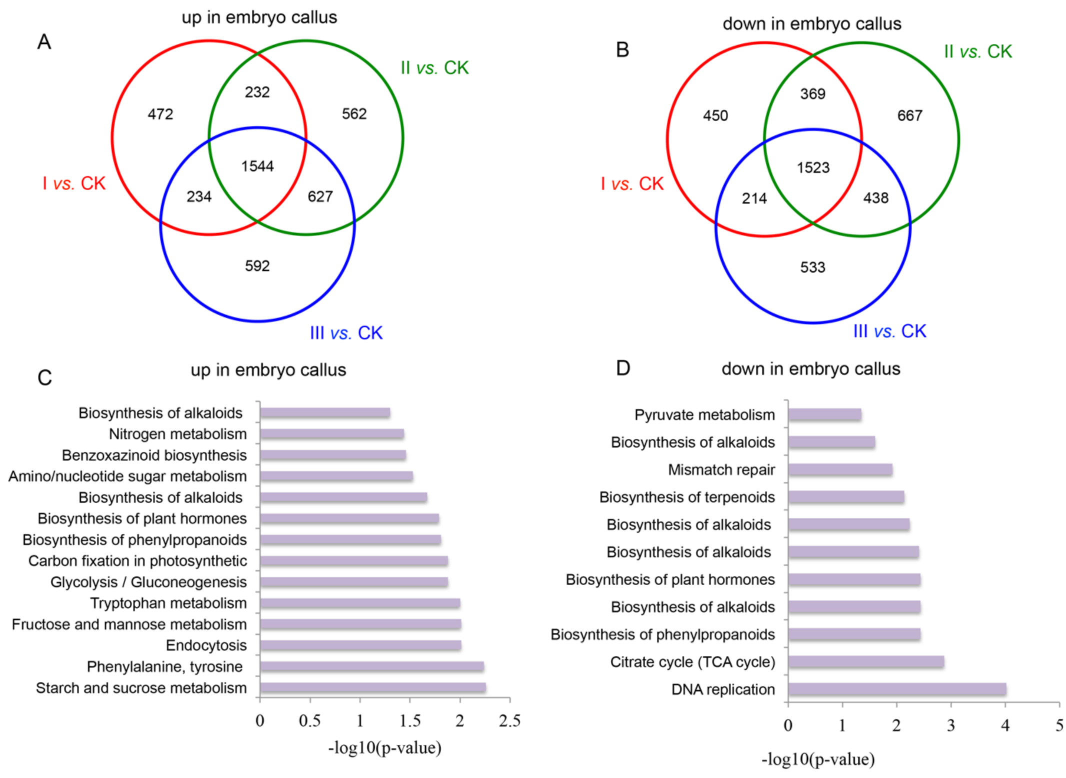

Figure S3. Venndiagram of 24-nt small RNA target DMRs and pathway results from DAVID. Venn diagrams display the intersection of target genes of 24-nt small RNAs that significantly positive correlated with DMRs.

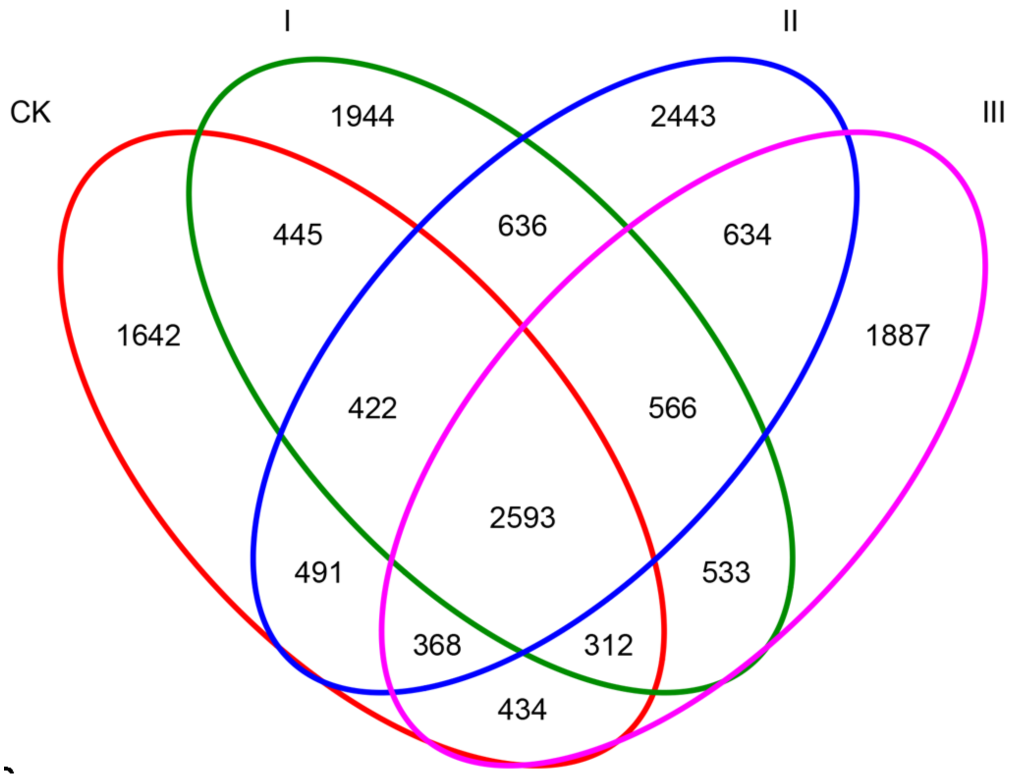

Supplement: Supplementary file 1 — Chromosomal distribution of DNA methylation read for each maize embryo sample. Each chromosomal was split in 10Kb windows. Fig. S2. Comparative and pathway analysis of DGE data. (A, B) Venn diagrams display the intersection of differentially expressed genes as determined by FDR < 0.001 and log2fold change >1 for genes A) up-regulated and B) downregulated in differentiated embryo compared to normal embryo (CK group) (I vs. CK, II vs. CK, III vs. CK). C, D) KEGG pathway analyses. Overrepresented KEGG pathways in genes up-regulated (C) and down-regulated (D) in differentiated embryos compared to CK group as calculated (P < 0.05) are shown. The x-axis displays the –log10 of the p-values calculated by DAVID (http://david.abcc.ncifcrf.gov). Fig. S3. Venndiagram of 24-nt small RNA target DMRs and pathway results from DAVID. Venn diagrams display the intersection of target genes of 24-nt small RNAs that significantly positive correlated with DMRs. (PDF 1136 kb) [file 12870_2017_1055_MOESM1_ESM.pdf]
